# Culture-induced recurrent epigenetic aberrations in human pluripotent stem cells

**DOI:** 10.1371/journal.pgen.1006979

**Published:** 2017-08-24

**Authors:** Uri Weissbein, Omer Plotnik, Dan Vershkov, Nissim Benvenisty

**Affiliations:** The Azrieli Center for Stem Cells and Genetic Research, Department of Genetics, Silberman Institute of Life Sciences, The Hebrew University, Jerusalem, Israel; UCLA, UNITED STATES

## Abstract

Human pluripotent stem cells (hPSCs) are an important player in disease modeling and regenerative medicine. Nonetheless, multiple studies uncovered their inherent genetic instability upon prolonged culturing, where specific chromosomal aberrations provide cells with a growth advantage. These positively selected modifications have dramatic effects on multiple cellular characteristics. Epigenetic aberrations also possess the potential of changing gene expression and altering cellular functions. In the current study we assessed the landscape of DNA methylation aberrations during prolonged culturing of hPSCs, and defined a set of genes which are recurrently hypermethylated and silenced. We further focused on one of these genes, testis-specific Y-encoded like protein 5 (*TSPYL5*), and demonstrated that when silenced, differentiation-related genes and tumor-suppressor genes are downregulated, while pluripotency- and growth promoting genes are upregulated. This process is similar to the hypermethylation-mediated inactivation of certain genes during tumor development. Our analysis highlights the existence and importance of recurrent epigenetic aberrations in hPSCs during prolonged culturing.

## Author summary

hPSCs were shown to acquire genetic aberrations during their growth in culture. The aberrations are non-random and positively selected, by altering multiple cellular phenotypes. Similarly to genetic mutations, epigenetic aberrations may also change gene expression levels, leading to altered cellular behaviors, as seen in tumors. In this study we focus on methylation changes, and we showed that there is a set of genes which are recurrently hypermethylated and silenced in high passage cells. One of these genes, *TSPYL5*, was shown to be downregulated by hypermethylation in multiple cancer types. We showed that upon its silencing in hPSC, differentiation genes and tumor-suppressor genes are downregulated, and pluripotency- and growth promoting genes are upregulated, which drive the positive selection. These results highlight another challenge faced by hPSC in regard to maintenance of intact gene expression program, and emphasize the role of epimutations described in cancer cells also to hPSCs.

## Introduction

Human pluripotent stem cells (hPSCs) play a major role in current genetic research and in future regenerative medicine, since they are capable of both self-renewal and differentiation into multiple cell types. The persistence for genetically intact cells is crucial in order to ensure safe hPSC-based treatments as well as reliable analysis of their characteristics. However, prolonged culturing of these cells, alongside their unique cell-cycle checkpoints, rapid cell cycle and high levels of replication stress, place them under constant selection pressures[1,2].

During prolonged culturing of PSCs, the cells might acquire genomic changes of variable magnitudes, ranging from full trisomies to copy number changes and point mutations in a process termed culture adaptation[3]. Among the recurrent genetic changes are trisomies of chromosomes 1,12,17 and X, and duplication of a specific region in chromosome 20[2–5]. These aberrations influence central properties such as cell growth, differentiation and susceptibility to apoptosis, and they are positively selected for and propagated until taking over the entire culture[2,6–8].

While gene expression levels are greatly affected by copy number variations or genetic mutations, they may also be modulated by epigenetic changes. Epigenetic mechanisms such as DNA methylation have crucial roles in maintaining gene expression patterns, hence, alterations in DNA methylation patterns may lead to erroneous gene expression. This phenomenon is well-documented in cancers, where epigenetic aberrations may contribute to tumor formation and growth. Global DNA hypomethylation is a common feature of both benign and malignant tumors[9]. It occurs mainly at repetitive sequences, coding sequences and promoters, leading to chromosomal instability, loss of imprinting and oncogenes activation. DNA hypermethylation occurs mainly in gene promoters leading to silencing of tumor suppressor genes[9,10].

Here we show that as in cancer, epigenetic aberrations also occur during culturing of pluripotent cells. We define a set of genes that are recurrently silenced due to DNA hypermethylation at CpG islands in their promotor. Specifically, we focus on testis-specific Y-encoded like protein 5 (TSPYL5), a gene which was previously shown to be hypermethylated and suppressed in multiple types of cancers[11–14]. We study the DNA methylation dynamics of TSPYL5 during culturing of hPSCs, and demonstrate its function in suppressing differentiation and upregulating growth-related genes.

## Results

### Identification of recurrent epigenetic changes in hPSCs

**Fig 1:**
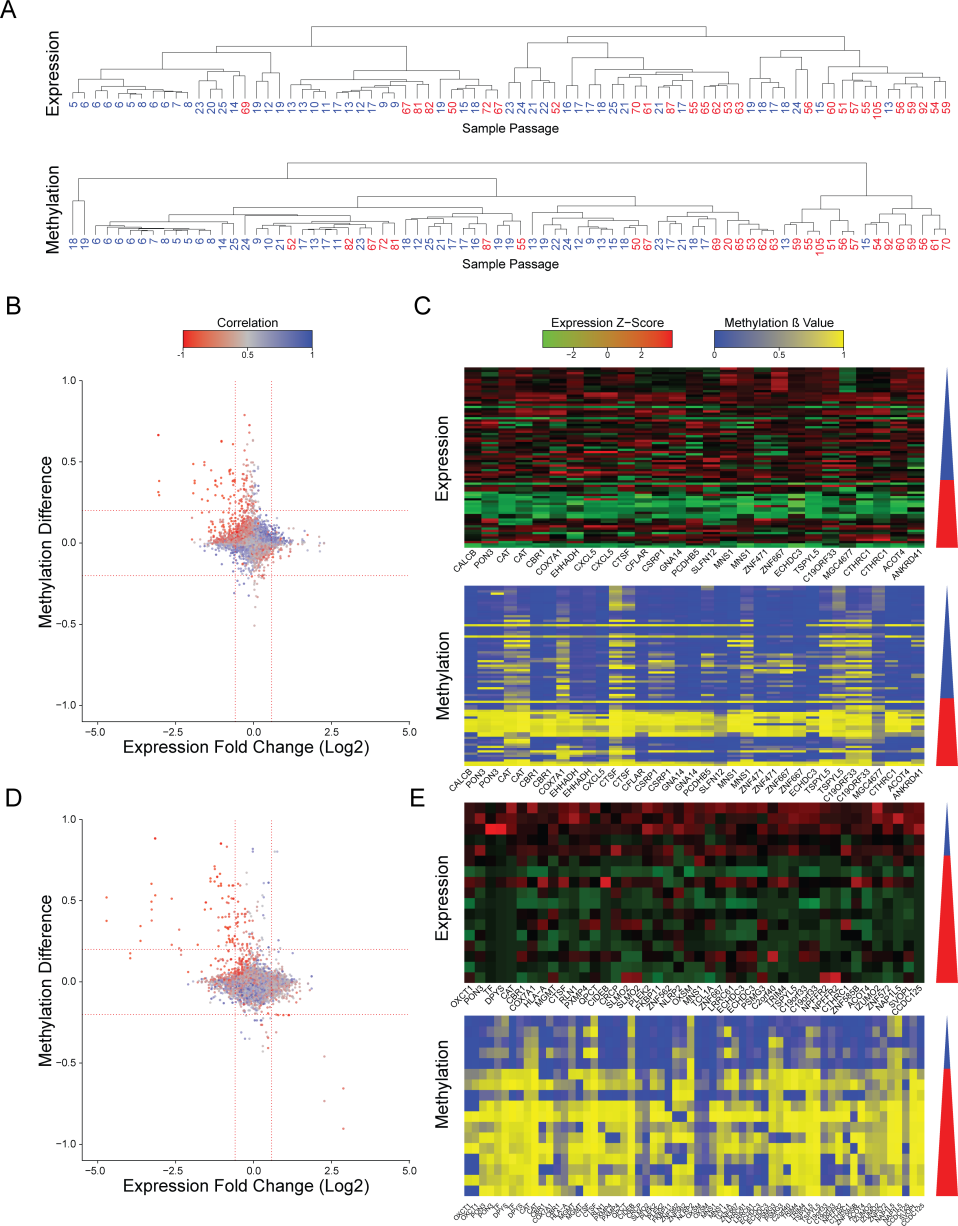
Changes in expression and methylation during prolonged culturing of hPSCs. **(A)** Hierarchical clustering of expression patterns (upper panel) and methylation patterns (lower panel) of the same samples. Number shown are the passage number of each sample. Clustering was performed using Pearson correlation and complete linkage. **(B)** Scatter plots of expression and methylation differences between high passage and low passage samples of data from Nazor et al[15]. Values represent the change between the averages of the high passage group and the low passage group. Colors indicate the correlation between the expression and methylation. Vertical red lines represent expression change of 1.5 fold. Horizontal red lines indicated methylation change of 0.2. **(C)** Expression and methylation heatmaps of the probes that showed hypermethylation and reduced expression at the high passage samples in data from Nazor et al[15]. The triangle to the right of each heatmap represents the increasing passage number, blue denotes the low passage samples and red denotes the high passage sample. **(D and E)** Same as (B and C) but for data from Mallon et al[17].

To evaluate whether recurrent epigenetic aberrations occur during culture adaptation of hPSCs, we have analyzed a database that assessed the DNA methylation state alongside gene expression levels in samples of different passage by methylation and expression arrays[15] (**S1 File**). Samples harboring large chromosomal aberrations, as detected by e-Karyotyping analysis[5,16] were discarded (**S1 Fig**). The remaining diploid samples were divided into two groups of low-passage cell lines (p25 or below) and high-passage cell lines (p50 or higher). Using this criterion, both groups have high number of samples with distinct separation regarding the passage numbers. Unbiased hierarchical clustering of the expression profiles showed that most high-passage samples clustered together, indicative of gene expression changes during culturing **(Fig 1A and S2 Fig)**. Methylation β values are measured as the ratio of the methylated bead intensity from the total locus intensity. Unbiased hierarchical clustering of the DNA methylation profiles also resulted in segregation of the samples by the passage number, hinting for unstable methylation during cell culture (**Fig 1A and S2 Fig**). This patterns of clustering were independent of the gender of the samples or the clustering methodology (**S3 and S4 Figs**). To gain a better understanding of this phenomenon we plotted the methylation β-value difference between the low- and high-passage samples for each probe, versus the expression change of the gene related to it. This analysis assess the differences in the average expression and methylation levels between the low- and high passage groups. We used a cutoff of methylation difference of 0.2 and expression fold change of 1.5 as this levels of change are statistically significant (less the 5% of the probes). Using this analysis, we detected 34 probes that were hypermethylated in the high-passage group, corresponding to 19 downregulated genes (**Fig 1B and 1C**). Importantly, there was a negative correlation between the expression and DNA methylation levels of these genes. (**Fig 1B and 1C**). These results were maintained even when removing the human induced pluripotent samples (hiPSCs) and using only samples of human embryonic stem cells (hESCs) (**S5 Fig**).

**Fig 2:**
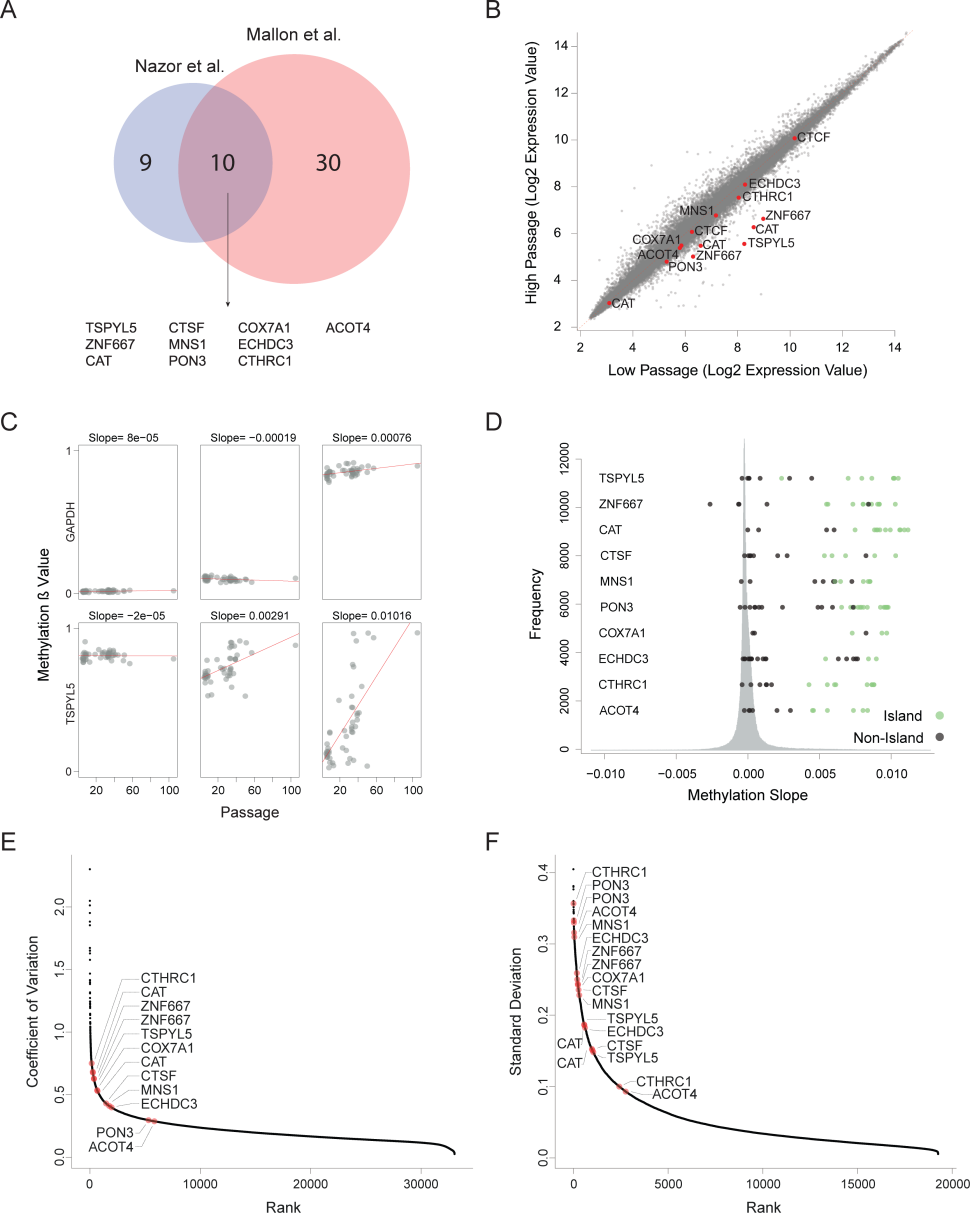
Recurrent hypermethylation during culturing occurs at regulatory CpG Islands. **(A)** Venn diagram defining 10 genes that undergo hypermethylation and silencing in both analyzed datasets. **(B)** Expression changes between high passage and low passage hESCs in large expression database of samples with given passage. Shown are the average values of each probe in each group. **(C)** Examples of methylation levels of six probes in different samples from different passages. In red are the linear regression lines. The slopes of the trend lines are presented above each graph. **(D)** Histogram describing the distribution of the methylation slopes of all the methylation probes. The methylation sloped of the probes of the 10 candidate genes are shown. **(E-F)** Variation in expression levels (E) and methylation values (F) in data sets of multiple hESCs without known passage number. Methylation probes shown are only those located in CpG islands.

To validate our results, we repeated the analysis using an independent database of matched expression and methylation profiles of pluripotent cell lines at different passages[17] (**S1 File**). Since this database is smaller, and it has the bias that all low passage samples are hiPSCs and all high passage samples are hESCs, we used this analysis as validation for the main analysis. Again, we detected a group of 68 methylation probes that were hypermethylated in the high-passage cell lines, correlating with reduced expression of the 40 corresponding expression probes (**Fig 1D and 1E**). Collectively, we have detected 10 candidate genes that were identified as hypermethylated and silenced in a passage-dependent manner by both assays (**Fig 2A,** random probability of 1.07×10^−21^)

For further validation of our findings, we assembled a large database of 68 expression profiles of hESCs with reported passage numbers from the Gene Expression Omnibus (GEO) database[18] (**S1 File**). We divided the samples to low-passage group (p25 or below) and a high-passage group (p25 and higher), and compared average expression levels between those groups. In this analysis we considered any sample above p25 as high passage sample since there were not enough samples with reported passage above p50. Out of the 10 genes detected by our previous analyses, 8 genes had probes showing statistically significant reduction in expression in the high-passage group compared with the low-passage group (FDR corrected Wilcoxon tests *P*<0.05, except ECHDC3 and CTCF) (**Fig 2B**).

To extend the validation of the DNA methylation data, we have utilized 48 samples analyzed using Illumina Infinium 450K Methylation BeadChips[15], enabling a much denser coverage (**S1 File)**. For each probe, we modeled the relationship between the methylation state and passage number by linear regression, and extracted the slope of the fitted line. (**Fig 2C**). Methylation slope near zero implies a stable methylation state during culturing, while positive or negative methylation slope values imply gain or loss of methylation during culturing. Compared with all the assayed probes (365,146 probes linked to a gene), most of the probes that correspond to CpG islands of the 10 candidate genes ranked very highly, among the 1% most hypermethylated CpGs. (**Fig 2D and S1 Table**). Probes located outside of the CpG Islands where relatively stable. These results support the notion that DNA hypermethylation of specific genes during culturing may turn off gene expression.

Since our data suggest that the expression and methylation states of certain candidate genes are not stable during prolonged culturing, we hypothesized that these genes will demonstrate high degree of variability in their methylation and expression levels relative to other genes. To examine our hypothesis, we collected a dataset of 126 expression microarray profiles and 46 Illumina 27k methylation arrays of hESCs (**S1 File**). Analysis of the variation levels revealed that the candidate genes are among the most variable genes in both expression and methylation (**Fig 2E and 2F**). Collectively, our analysis pointed to 10 genes whose silencing by aberrant hypermethylation is selected during prolonged culturing of hPSCs.

### Hypermethylation and downregulation of *TSPYL5* in hPSCs during prolonged culturing

**Fig 3:**
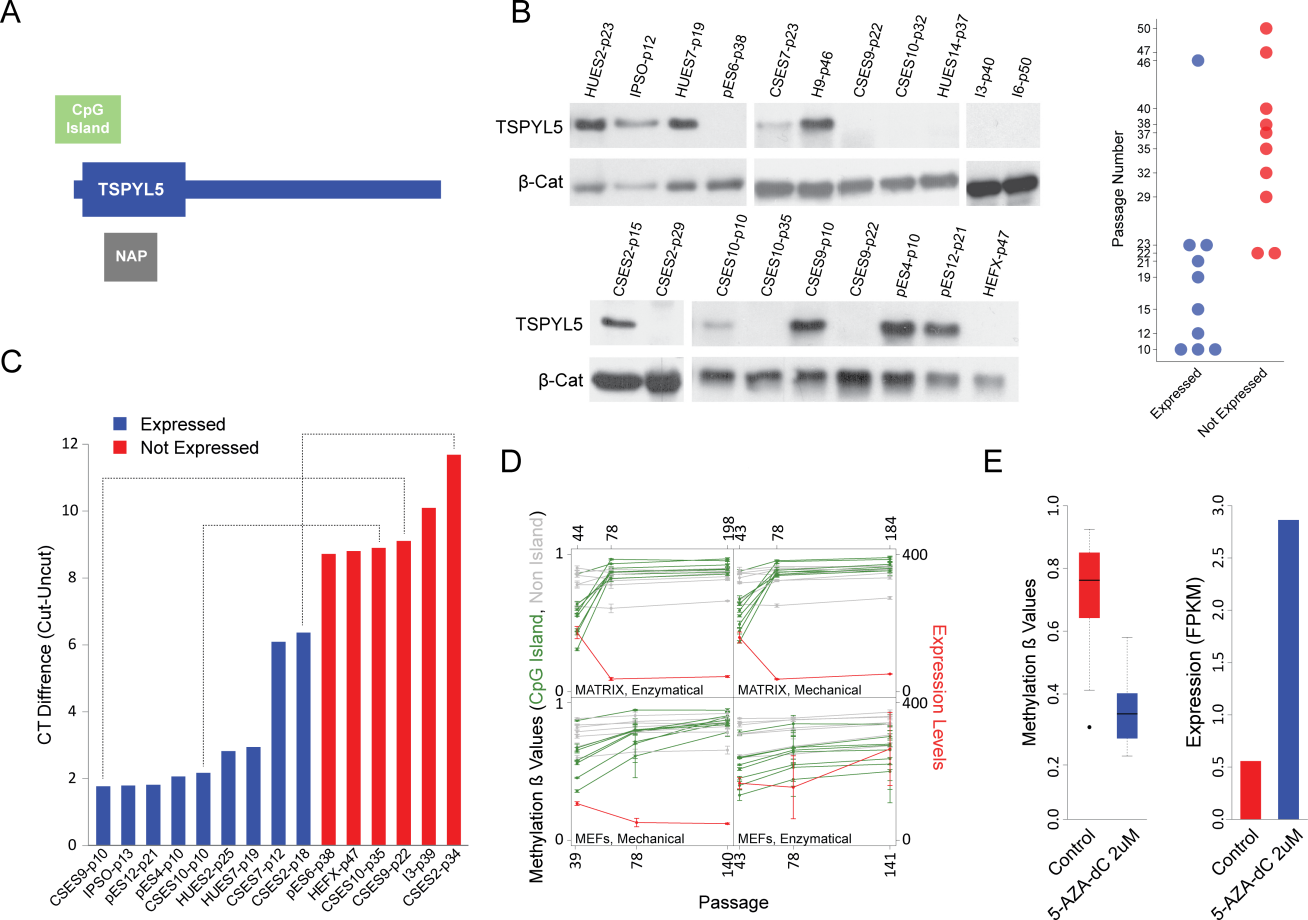
*TSPYL5* undergoes hyper methylation and silencing in culture. **(A)** Schematic representation of *TSPYL5* gene. **(B)** Western blot analysis for TSPYL5 of samples from multiple hPSCs cell lines at different passages. The plot at the right panel represents the TSPYL5 expression state of each sample as detected in the western blot analysis. **(C)** Analysis of methylation levels using the methylation sensitive enzyme McrBC. Shown are differences between quantitative-PCR (qPCR) results of digested and undigested DNA. Blue samples express *TSPYL5* while red samples do not. Samples of the same cell line are connected with dashed line. **(D)** *TSPYL5* expression and methylation changes during continuous passaging of the WA09 cell line. Samples were grown either on extracellular matrix or on MEFs, and passaged mechanically or enzymatically. **(E)** Changes in *TSPYL5* Expression and methylation levels in pES6 cells after 5 days treatment with 5-AZA-dC.

Out of the 10 hypermethylated genes, we focused on *TSPYL5* (**Fig 3A**) since it was shown to be hypermethylated in various types of cancers including glioma[13], glioblastoma[13], astrocytomas[13], gastric cancer[11], esophageal squamous cell carcinomas[14], hepatocellular carcinoma[19] and endometrial cancer[20]. In some of these tumors, hypermethylation was correlated with reduced *TSPYL5* expression[11,13]. Furthermore, re-expression of *TSPYL5* in glioma and gastric cell lines, in which the endogenous *TSPYL5* promoter was shown to be methylated, suppressed cell growth in culture[11,13]. This gene was also shown to be involved in resistance to γ-radiation in lung adenocarcinoma cells[12]. The observed hypermethylation in multiple types of cancers suggests that TSPYL5 may play a role in cell growth regulation and survival.

To understand the role of TSPYL5 in hPSCs, we have analyzed the pattern of *TSPYL5* gene expression during early human development, from zygote to early blastocyst and low-passage hESCs, using microarray data[21]. Comparison of *TSPYL5* expression to known maternal genes (*FIGLA* and *ZP2*) shows that *TSPYL5* is a zygotic gene that is actively expressed in early stage hESCs (**S6A Fig**). Additionally, *TSPYL5 is* highly expressed in naïve hPSCs[22], which represent the ground state of pluripotency, and during the transition from primed to naïve state it is induced by 10 folds (**S6B Fig**). These results suggest that *TSPYL5* is normally expressed in hESCs, and that its disappearance during prolonged culturing is aberrant.

To confirm these observations, we examined TSPYL5 protein levels in 12 different hPSC lines from various passages. Indeed, the majority of low-passage cell lines expressed TSPYL5, whereas high-passage samples did not (**Fig 3B**). Importantly, in the cell lines CSES2, CSES9 and CSES10, expression of TSPYL5 was detected at low passage, but not at latter passages (**Fig 3B**). To determine if silencing of *TSPYL5* is indeed due to hypermethylation at the promoter CpG island, we used the McrBC restriction enzyme, whose activity depends on methylated CpG sites. Cell lines that expressed TSPYL5 protein showed hypomethylation in its DNA sequences (**Fig 3C**). To further support the relation between DNA methylation and expression of *TSPYL5*, we re-analyzed expression and methylation data of the WA09 hESC line grown continuously for more than 100 passages[23]. This analysis showed that, *TSPYL5* was indeed expressed at the beginning of the experiment and subsequently silenced after intensive passaging, accompanied by hypermethylation of the CpG island (**Fig 3D**). Overall, our results demonstrate that the methylation and silencing of TSPYL5 are not limited to specific cell lines, but is a general culture-induced process.

To show that the methylation state of the promoter of *TSPYL5* determines its expression, we treated the TSPYL5-non-expressing cell line pES6 with the demethylating agent 5-aza-2'-deoxycytidine (5-aza-dC). As expected, the treatment caused massive demethylation at the *TSPYL5* locus, which was sufficient to reactivate its expression, pointing to the importance of methylation in the regulation of this gene (**Fig 3E**).

### TSPYL5 silencing modifies expression of differentiation, pluripotency and growth related genes

**Fig 4:**
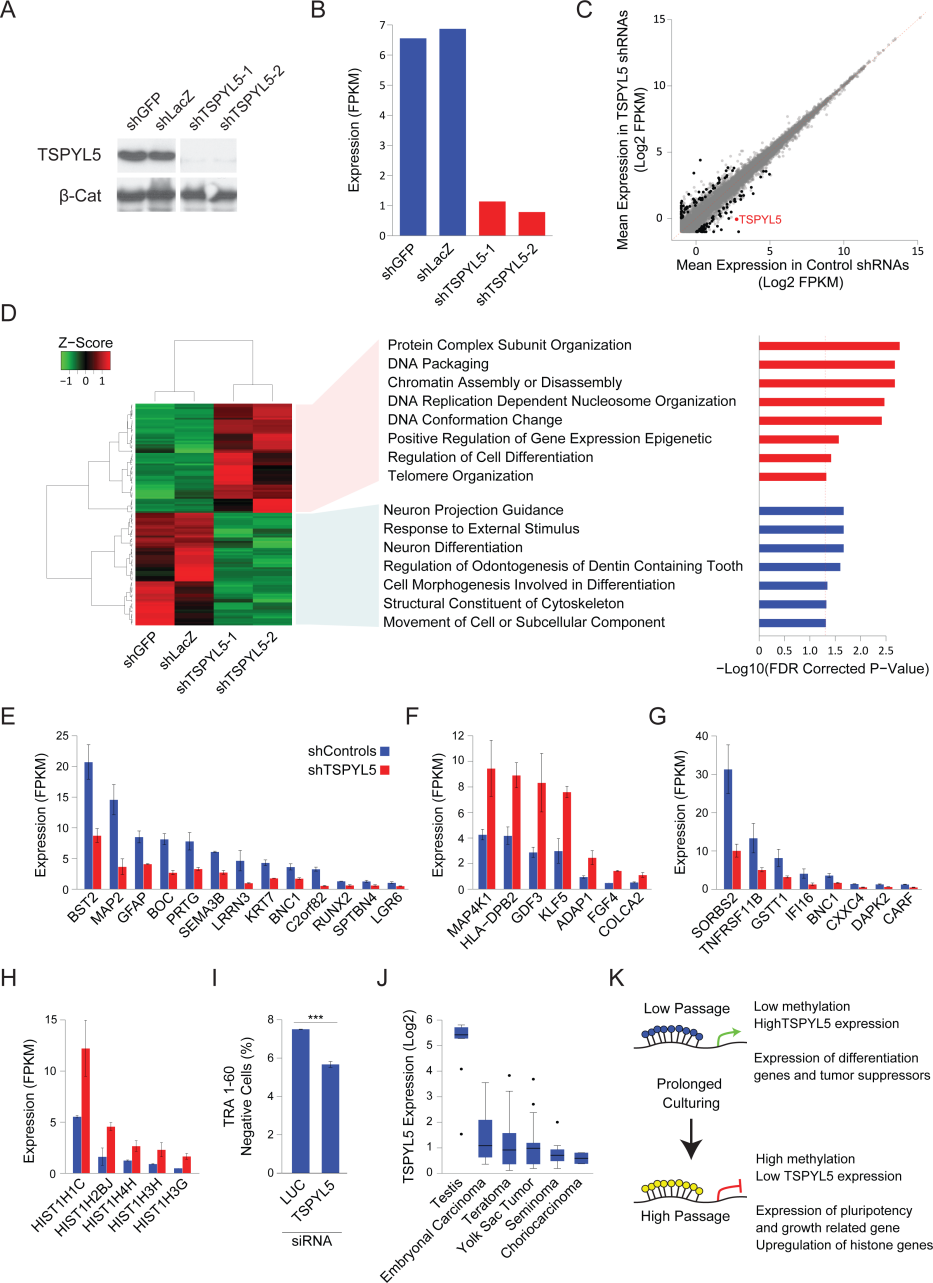
Gene expression analysis upon *TSPYL5* knockdown shows its importance in differentiation. **(A-B)** Western blot analysis (A) and FPKM expression levels (B) for TSPYL5 for CSES7 cells after knockdown with shRNAs or control constructs. **(C)** Scatter plot of gene expression levels of sh*TSPYL5* cells versus controls. Values are the mean of both repeats. Genes marked in black are those with two folds change, and both repeats have at least 1.5 folds change. **(D)** Heatmap of the genes that showed significant change. For the up- or down-regulated genes, GO Enrichment categories, analyzed in GSEA are shown. **(E-H)** Bar plots of expression levels of differentiation genes (E), pluripotency and growth related genes (F), tumor suppressor genes (G), and histone genes (H). **(I)** Percentage of TRA-1-60 negative cells after treatment with siRNA for Luciferase (used as control) or TSPYL5. Statistical significance was analyzed with Student’s t-test; ***p<0.001 **(J)** *TSPYL5* expression levels in multiple types of GCTs and testis as a control. **(K)** A model describing the observed changes in methylation during prolonged culturing of hPSCs.

TSPYL5, which is located on chromosome 8, was suggested to contain a nucleosome assembly protein (NAP) domain[11], and was shown to bind gene promoters[24] and may thus affect gene expression[24,25]. To examine the effects of *TSPYL5* silencing in culture we used shRNAs to knockdown *TSPYL5* expression in a *TSPYL5-*expressing cell line (**Fig 4A**). Analysis of gene expression by RNA sequencing showed the expected downregulation of *TSPYL5* in the knockdown cell lines (**Fig 4B**). Then, we selected genes whose mean expression changed after *TSPYL5* knockdown, identifying 126 differentially expressed genes (fold change between averages>2, and both repeats have fold change>1.5) (**Fig 4C**). Gene set enrichment analysis (GSEA) revealed a significant enrichment for genes involved in differentiation among the downregulated genes (**Fig 4D and 4E and S2 Table**), while the upregulated genes were enriched for chromatin-related genes (**Fig 4D and S2 Table**). The upregulated gene set also included known genes related to pluripotency and growth, such as *KLF5*, *FGF4* and *GDF3* (**Fig 4F**), and 8 downregulated genes were known tumor suppressors (**Fig 4G**). Interestingly, five of the upregulated genes were histone coding genes (**Fig 4H**).

To support the notion that the downregulation of many differentiation-related genes upon TSPYL5 silencing may affect the extent of spontaneous differentiation, we knockdown TSPYL5 with siRNAs in low-passage TSPYL5-expressing cell line. We then assessed the percentage of differentiated cells by immunostaining for the pluripotency marker TRA-1-60, and analyzing the cells with fluorescence-activated cell sorting (FACS). In concordance with the transcriptomic analysis, knockdown of TSPYL5 results in less spontaneous differentiation (**Fig 4I**).

Lastly, we asked whether TSPYL5 silencing also occurs in germ cell tumors (GCT), which are tumors of germ cells origin that initiate from primordial germ cells at different developmental stages[26]. For this analysis, we assembled microarray expression data from two independent studies of different GCTs and normal testis (**S7 Fig**). Similar to the detected silencing in hPSCs grown in culture, *TSPYL5* expression was reduced in all GCTs samples compared to normal testis control (**Fig 4J**), suggesting that TSPYL5 silencing can have advantage also *in-vivo*.

## Discussion

The identification of specific chromosomal aberrations that are recurrently acquired and propagated in hPSC cultures, have highlighted the importance of frequent examination of the genetic integrity of these cells. Our work demonstrates that the same approach may also apply for epigenetic aberrations, were a defined set of loci gain DNA methylation that reduces gene expression. These stochastic changes are propagated by positive selection forces conferred by alterations in pathways that regulate cellular properties such as differentiation and pluripotency, as we show here for *TSPYL5* (**Fig 4K**). Further research is needed in order to assess the importance of this phenomenon to downstream applications of hPSCs.

The growth of cells in culture has many similarities to cancerous processes. Both processes are characterized by accumulation of mutations that provide a growth advantage to the aberrant cells. Genetic mutations of different magnitudes, ranging from the chromosome scale to single nucleotides, have been widely analyzes in hPSCs[2,6,7,27]. Here we showed that epimutations, in the form of aberrant DNA methylation, could also occur during culturing, altering known pathways that are often modulated during tumorigenesis.

While the mechanisms enabling the acquisition of aberrant DNA methylation requires further study, it is important to note that hPSCs had been reported to have defects in the maintenance of intact methylation patterns regarding various processes. Loss of the monoallelic patterns of expression at imprinted loci was repeatedly documented in hPSC lines[15,28,29]. This altered pattern of expression was attributed to aberrant methylation[15,28,29]. In addition, X-chromosome inactivation, which is initiated by the expression of *XIST* RNA and maintained by DNA methylation and heterochromatinization, was shown to vary among female hPSC cell lines[30,31]. This phenotype was shown to be related to changes in DNA methylation in the X chromosome[15]. The methylation instability in hPSCs can be attributed to the dynamic nature of the methylation maintenance mechanisms in pluripotent cells[32], coupled with the expression of the de-novo methyltransferases DNMT3A and DNMT3B[33].

In our large-scale analysis, we have suggested 10 candidate genes that are frequently undergo epigenetic silencing during passaging. Although our analysis was comprehensive, there may be additional genes that become silenced during culturing, but are not represented in the platforms used for analyzing DNA methylation and gene expression levels. Using analysis of bisulfite sequencing together with RNA-seq may be beneficial in this regard.

We chose to focus on the role of *TSPYL5* in culture adaptation due to the multiple reports showing hypermethylation and silencing of *TSPYL5* in various tumors. Through their interaction with chromatin, NAP domain proteins such as TSPYL5 were shown to alter gene expression, apoptosis and the cell cycle[25]. Indeed, we demonstrated that *TSPYL5* silencing may cause reduction in expression of differentiation-promoting genes and tumor suppressors, and upregulation in pluripotency- and growth-related genes. These changes can translate into less spontaneous differentiation in culture. Thus, our data suggest positive selection forces when *TSPYL5* is silenced, and support its relevance to tumor progression.

Lastly, we showed that *TSPYL5* is also downregulated in GCT samples. It was shown that similar chromosomal aberrations are gained in tumors and in cultured stem cells of the same tissue[34]. The most prominent example is the enrichment of trisomy 12 in both hPSCs and GCT. Here, we show that *TSPYL5* expression is markedly reduced also in both high-passage hPSCs and GCTs, supposedly due to common selection forces. Overall, we demonstrate an additional epigenetic maintenance challenge faced by hPSCs grown in culture, and emphasize its potential importance in regard to tumorigenesis.

## Materials and methods

### Microarray expression and methylation profiles analysis

Published expression microarray profiles were downloaded from the Gene Expression Omnibus database[18] and RMA normalized using Affymetrix Expression Console. Expression values below a certain threshold were raised to that fixed threshold level to reduce noise of non-expressing genes. The original dataset contains 88 samples with methylation and expression arrays, of cell lines passages < = 25 and > = 50. 11 samples were excluded from the analysis due to large chromosomal aberration or noisy transcriptome as determined by e-Karyotyping[16]. None of the samples contained deletion or amplifications of chromosome 8. Methylation profile were downloaded as β values from the GEO database. Methylation β values of each probe from Nazor et al. were obtained by range-scaling to control samples of unmethylated, completely methylated and half-methylated-DNA[15].

For detection of methylation and expression changes during culturing only samples that had both methylation and expression data where included in this analysis. Sample were divided into two groups by their passage, a group of samples below or equal to passage 25 and a group of samples of passage equal or above 50. Probes on the sex chromosomes where discarded as well as genes unexpressed in more than 80% of the samples in both groups. The expression fold between the two groups was calculated by dividing the median values. Methylation difference where calculated by subtracting the ß-values. The correlation between the expression and methylation was also calculated for each probe. Genes were considered hypermethylated and silenced if the changes in expression were above 1.5 folds, and methylation differences were above 0.2.

For the analysis of multiple expression profiles collected from different studies, samples were divided into low passage samples (passage 25 or lower) and high passage samples (passage 25 and more). In this analysis we were less restrictive in the high passage cutoff due to lack of samples with very high passage number.

For the analysis of Infinium 450K Methylation beadChips (Illumina), for each probe we preformed linear regression between the methylation values and the passage numbers and extracted the slope values. This slope values were considered as an estimate for the methylation change over time in culture.

### Cell culture

hESCs were cultured on mouse embryonic fibroblast (MEF) treated with mitomycin-C. Growth medium contained KnockOut Dulbecco's modified Eagle's medium (Gibco-Invitrogen, CA) supplemented with 15% KnockOut-SR (Gibco-Invitrogen, CA), 1mM glutamine, 0.1mM ß-mercaptoethanol (Sigma-Aldrich, MO), 1% nonessential amino acids stock (Gibco-Invitrogen, CA), penicillin (50units/ml), streptomycin (50μg/ml), and 8ng/ml FGF2 (Gibco-Invitrogen, CA). 10μM ROCK inhibitor (Y27632) was supplemented to the medium in the first 24h after thawing cells. Cells were passaged using short treatment with trypsin or trypsin-EDTA (Biological Industries, Beit Haemek, Israel) maintaining the cells as clumps of multiple cells.

### Methylation analysis by McrBC digestion

The analysis is based on previously described method[35]. Genomic-DNA was extracted using NucleoSpin Tissue kit (Macherey-Nagel). 500ng of genomic-DNA was digested in 30μl reaction using McrBC enzyme (NEB) for 3h at 37°C and hit-inactivated at 65°C for 20min. As a control, parallel reaction without McrBC was performed. The restriction products diluted to 90μl and 5μl served as a temple for Real-time PCR analysis with primers surrounding the CpG island promoter region. Product could be amplified only if there was no restriction, meaning all the amplicon was unmethylated. qPCR was performed using PerfeCTa SYBR Green SuperMix (Quantabio) with the following conditions: 2min 50°C, 10min 95°C, 45 cycles of 15sec 95°C, 1min 65°C. Primers used are CCCCGAGACTCTGGTACTGT and GAGTCCGCGCGAGATGG.

### 5-Aza-2'-deoxycytidine treatments

pES6 cells were plated in a density of 100,000 cells per well in a six well plate and cultured for 24h before the beginning of the treatment. 5-Aza-dC (Sigma-Aldrich) was administered for 5 days in a final concentration of 2μM. Cell media was exchanged every day, supplemented with fresh 5-aza-dC. At the end of the experiment, genomic-DNA was extracted and analyzed by Infinium 450K Methylation beadChips (Illumina) and RNA was extracted and analyzed by RNA-Sequencing.

### DNA methylation analysis by methylation arrays

DNA methylation analysis was performed on genomic DNA using Infinium 450K Methylation beadChips (Illumina) following the Infinium HD Methylation Protocol as was previously described[28]. Data was processed and normalized by using subset-quantile within array normalization (SWAN) and adjusted for batch effects using the R package ChAMP (version 1.4.0).

### Western-blot analysis

Polyacrylamide gel (10%) was used for protein separation. Gels were transferred to a nitrocellulose membrane, and antibody hybridization and chemiluminescence were performed according to standard procedures. The primary antibodies used in this analysis were rabbit anti-TSPYL5 (N15) sc98186 (Santa Cruz Biotechnology) and mouse anti-ß-Catenin (AC-15) sc69879 (Santa Cruz Biotechnology). Horseradish peroxidase-conjugated anti-mouse and anti-rabbit secondary antibodies were obtained from Jackson ImmunoResearch Laboratories.

### TSPYL5 knockdown experiments

For stable knockdown of TSPYL5 we used shRNAs plasmids obtained from the MISSION shRNAs collection (Sigma-Aldrich). *TSPYL5* constructs used are TRCN0000322652 and TRCN0000322581. LacZ and GFP shRNA constructs used were TRCN0000072224 and TRCN0000072194, respectively. Plasmids were co-transfected together with packaging plasmids using polyethylenimine (PEI) into 293T cells. The medium was filtered through 45μM filter and stored at -80°C. CSES7 cells were infected with the lentiviruses during splitting. Puromycin selection (0.34μM) was initiated 2 days after infection. Surviving cells were pulled together.

For Transient knockdown, we used MISSION esiRNA (Sigma-Aldrich) for TSPYL5 (EHU046361) and Renilla Luciferase (EHURLUC). siRNAs were reverse-transfected into low-passage CSES10 cells. For single well of 12 well plate, 30-50nM siRNA was mixed with 1.4μl of DharmaFECT-1 transfection reagent (Dharmacon) in 250μl Opti-MEM I reduced serum media (Thermo Fisher Scientific) for 30 minutes. The transfection mix was then added to 40000 cells plated on Matrigel-coated plates (Corning) cultured in mTeSR medium (STEMCELL Technologies). The cells were grown for 3 days with fresh mTeSR medium supplemented every 24 hours.

### RNA extraction, sequencing and analysis

Total RNA was extracted using NucleoSpin RNA Plus kit (Marcherey-Nagel). RNA integrity (RIN>9) was validated using Bioanalyzer (Agilent Technologies). mRNA was enriched by Poly-A selection and sequencing libraries were prepared using TruSeq RNA Library Prep Kit v2 (Illumina). Single-end 85bp sequencing was performed using Illumina Next-Seq500.

Raw sequencing reads were aligned to HG19 reference genome using TopHat2[36] and Cufflinks was used to obtain normalized FPKM values for each sample[37]. Expression threshold was defined as 0.5, elevating genes with a lower expression to this level. To select genes with changed expression levels between the TSPYL5 shRNA and the controls, we set a threshold of two folds change. We also required that both repeats would have fold change of 1.5 over the two controls. GO enrichment analysis for upregulated or downregulated genes was performed in the Broad Institute’s Gene Set Enrichment Analysis (GSEA) tool (http://software.broadinstitute.org/gsea/)[38]

### TRA-1-60 immunocytochemistry

Cells were trypsinized using TrypLE Select (Thermo Fisher Scientific) in cold PBS containing 10% KSR, centrifuged and resuspended in 200 μl PBS containing 10% KSR. Staining was performed with PE-conjugated mouse anti-human TRA-1-60 antibody (1:40, BD Biosciences) for 30 minutes at 4°C with rotation. The cells were then washed with PBS containing 10% KSR, centrifuged at 300g at 4°C and resuspended in PBS with 10% KSR. Stained cells were filtered through a 70-μm cell strainer and analyzed in BD FACSAria III for the proportion of TRA-1-60-negative and positive cells.

### Statistics

All analyses were performed in R statistical software (http://www.r-project.org/). Unsupervised Hierarchical clustering analyses were performed using Pearson correlation or Euclidean distances and complete linkage. To test statistical significance of the clustering, the two main branched were analyzed for the proportion of low- and high passage samples by two-tailed Fisher’s exact test.

## Supporting information

S1 Fig E-Karyotyping analysis of all the samples from Nazor et al. that were used in the analysis. (TIF)

S2 Fig Analysis of the hierarchical clustering presented in Fig 1A. The proportion of low- and high passage samples in the two main branches of the trees are presented. P-values were calculated with Fisher’s exact test; *p<0.05, **p<0.01, ***p<0.001.
(TIF)

S3 Fig Assessment of the importance of the gender to the hierarchical clustering. **(A)** Hierarchical clustering of expression and methylation patterns of data from Nazor et al. according to the gender of the samples. Number shown are the passage number of each sample. Clustering was performed using Pearson correlation and complete linkage. **(B)** Analysis of the hierarchical clustering presented in A. The proportion of low- and high passage samples in the two main branches of the trees are presented. P-values were calculated with Fisher’s exact test; NS, not significant, *p<0.05, **p<0.01, ***p<0.001,
(TIF)

S4 Fig Euclidean distances hierarchical clustering of Nazor et al. data. **(A)** Hierarchical clustering of expression and methylation patterns of data from Nazor et al. Number shown are the passage number of each sample. Clustering was performed using Euclidean distances and complete linkage. **(B)** Analysis of the hierarchical clustering presented in A. The proportion of low- and high passage samples in the two main branches of the trees are presented. P-values were calculated with Fisher’s exact test; *p<0.05, **p<0.01, ***p<0.001.
(TIF)

S5 Fig Changes in expression and methylation during prolonged culturing of hESCs. Scatter plots of expression and methylation differences between high passage and low passage samples of data from Nazor et al[15]. Here, only hESCs were included in the analysis. Values represent the change between the averages of the high passage group and the low passage group. Colors indicate the correlation between the expression and methylation. Vertical red lines represent expression change of 1.5 fold. Horizontal red lines indicated methylation change of 0.2.
(TIF)

S6 Fig *TSPYL5* is expressed in early embryos and naïve hESCs. **(A)** Expression of two maternally deposit genes (*FIGLA* and *ZP2*), *NANOG* and *TSPYL5* in different stages during early human development **(B)** *TSPYL5* expression levels in hPSCs grown either with primed medium or with naïve medium[22].
(TIF)

S7 Fig Principle component analysis (PCA) of expression data form multiple samples of different GCTs. (TIF)

S1 Table Methylation slope ranking of the candidate genes. The methylation slope ranking of the 10 candidate genes, out of the total 365,146 probes, according to their position along the gene. High-ranking means high positive methylation slope, low ranking means negative methylation slope.
(DOCX)

S2 Table GSEA enrichment category for the genes up regulated or down regulated after *TSPYL5* knockdown. (DOCX)

S1 File List of published samples that were used in this study. (XLSX)
