# Supplementary figures and images for: Culture-induced recurrent epigenetic aberrations in human pluripotent stem cells
Source: PLoS Genet. 2017 Aug 24;13(8):e1006979. doi: 10.1371/journal.pgen.1006979 (PMC5587343; doi:10.1371/journal.pgen.1006979)

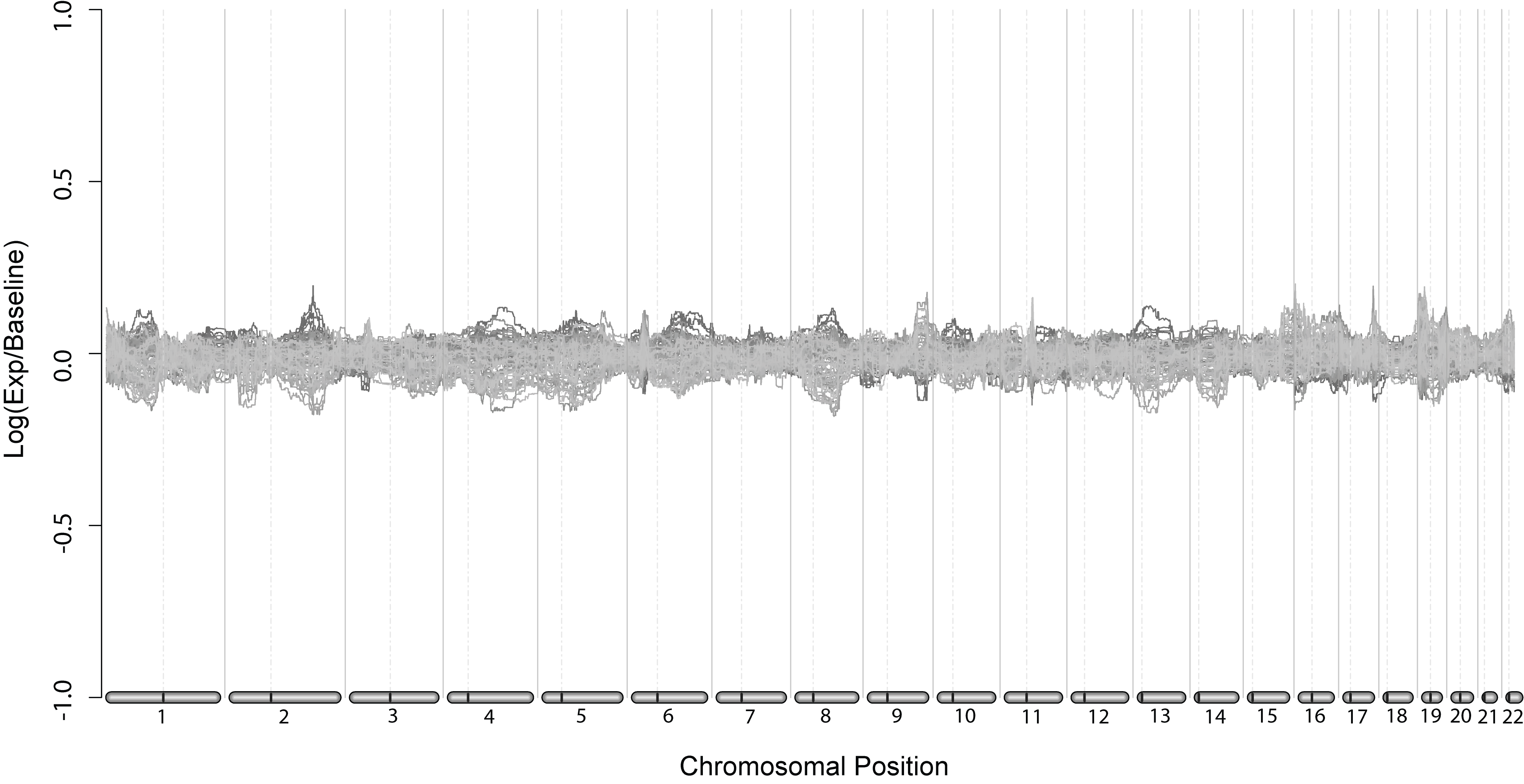

Supplement: S1 Fig — (TIF) [file pgen.1006979.s001.tif]

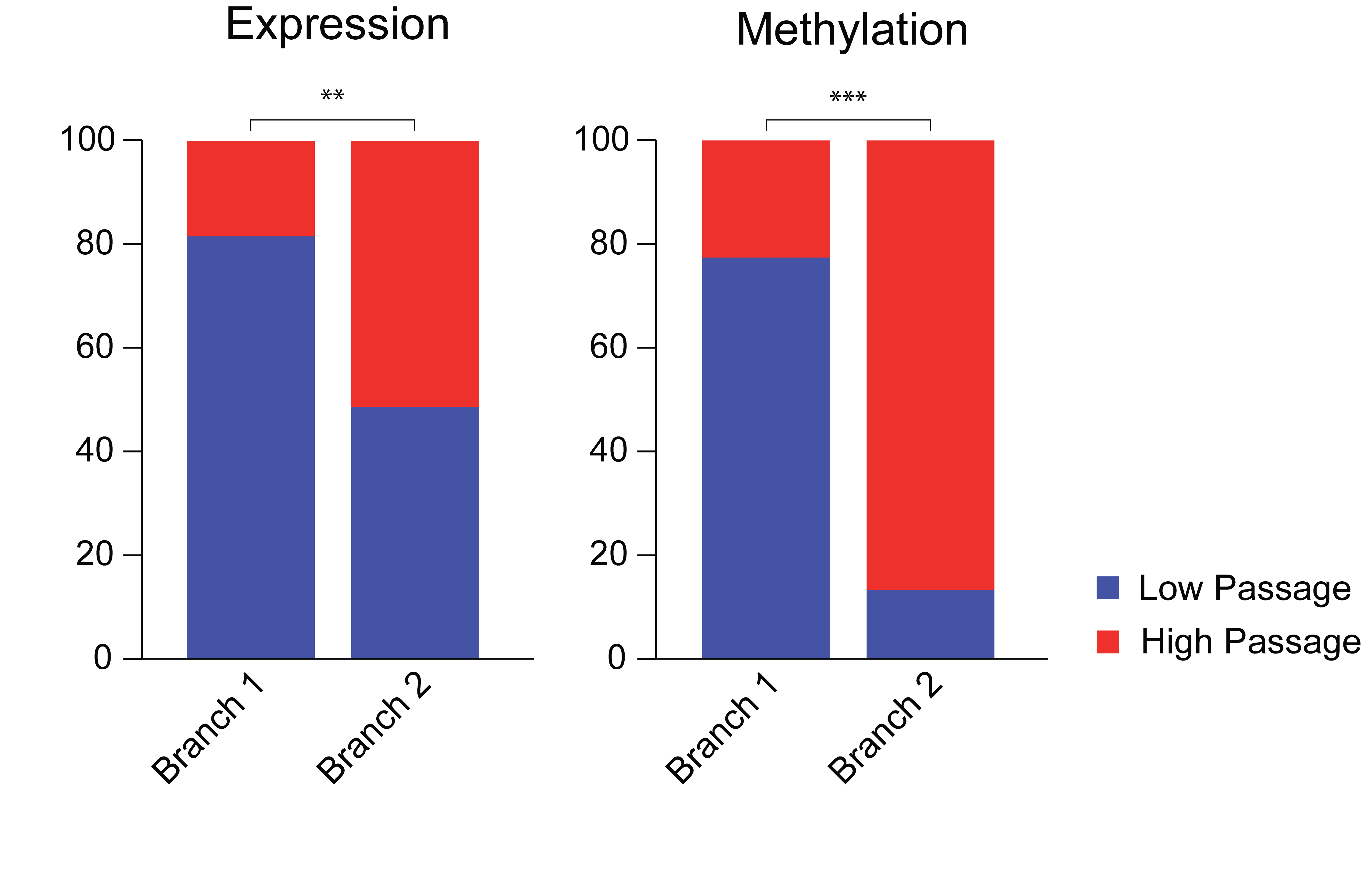

Supplement: S2 Fig — The proportion of low- and high passage samples in the two main branches of the trees are presented. P-values were calculated with Fisher’s exact test; *p<0.05, **p<0.01, ***p<0.001. (TIF) [file pgen.1006979.s002.tif]

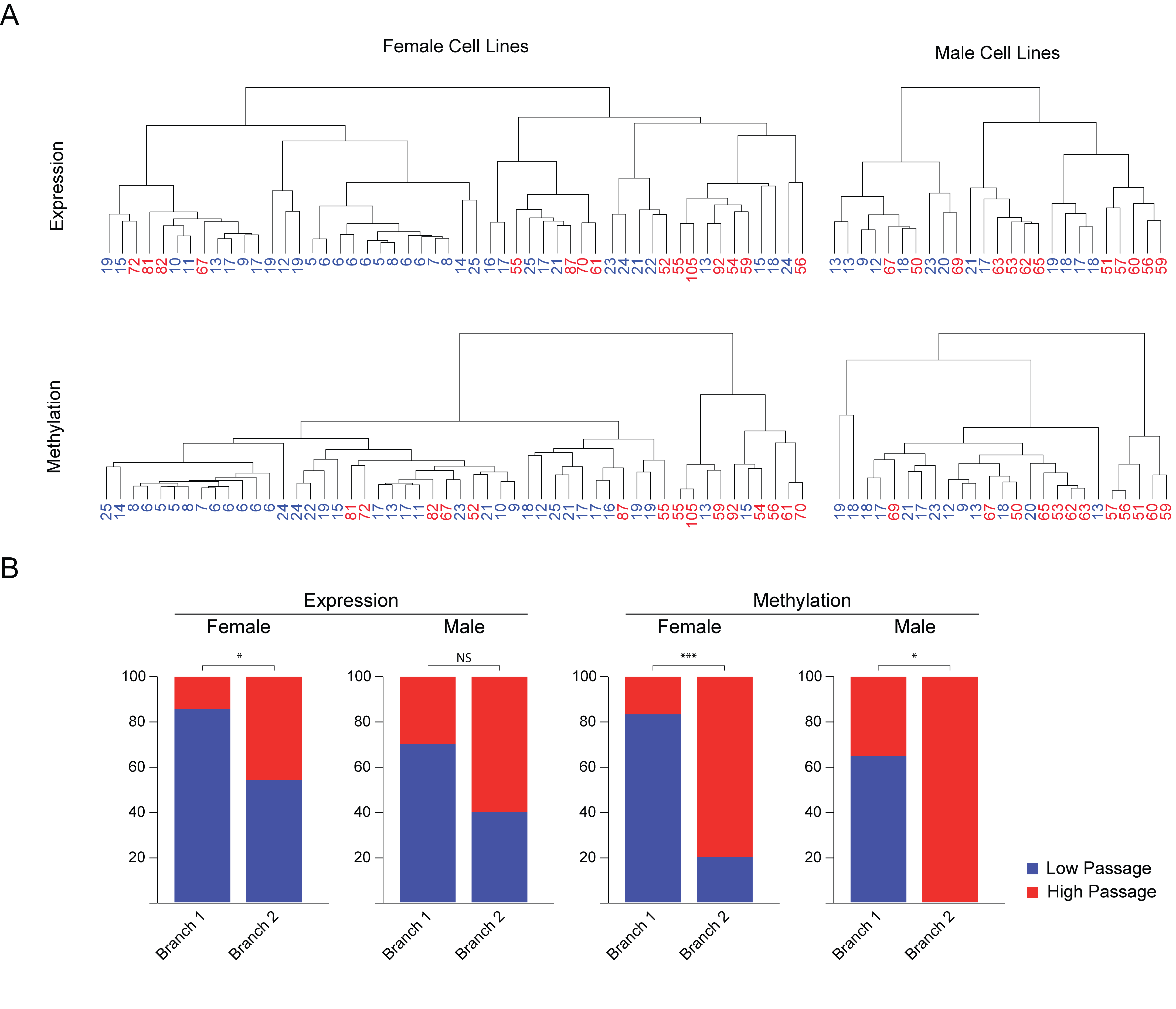

Supplement: S3 Fig — (A) Hierarchical clustering of expression and methylation patterns of data from Nazor et al. according to the gender of the samples. Number shown are the passage number of each sample. Clustering was performed using Pearson correlation and complete linkage. (B) Analysis of the hierarchical clustering presented in A. The proportion of low- and high passage samples in the two main branches of the trees are presented. P-values were calculated with Fisher’s exact test; NS, not significant, *p<0.05, **p<0.01, ***p<0.001, (TIF) [file pgen.1006979.s003.tif]

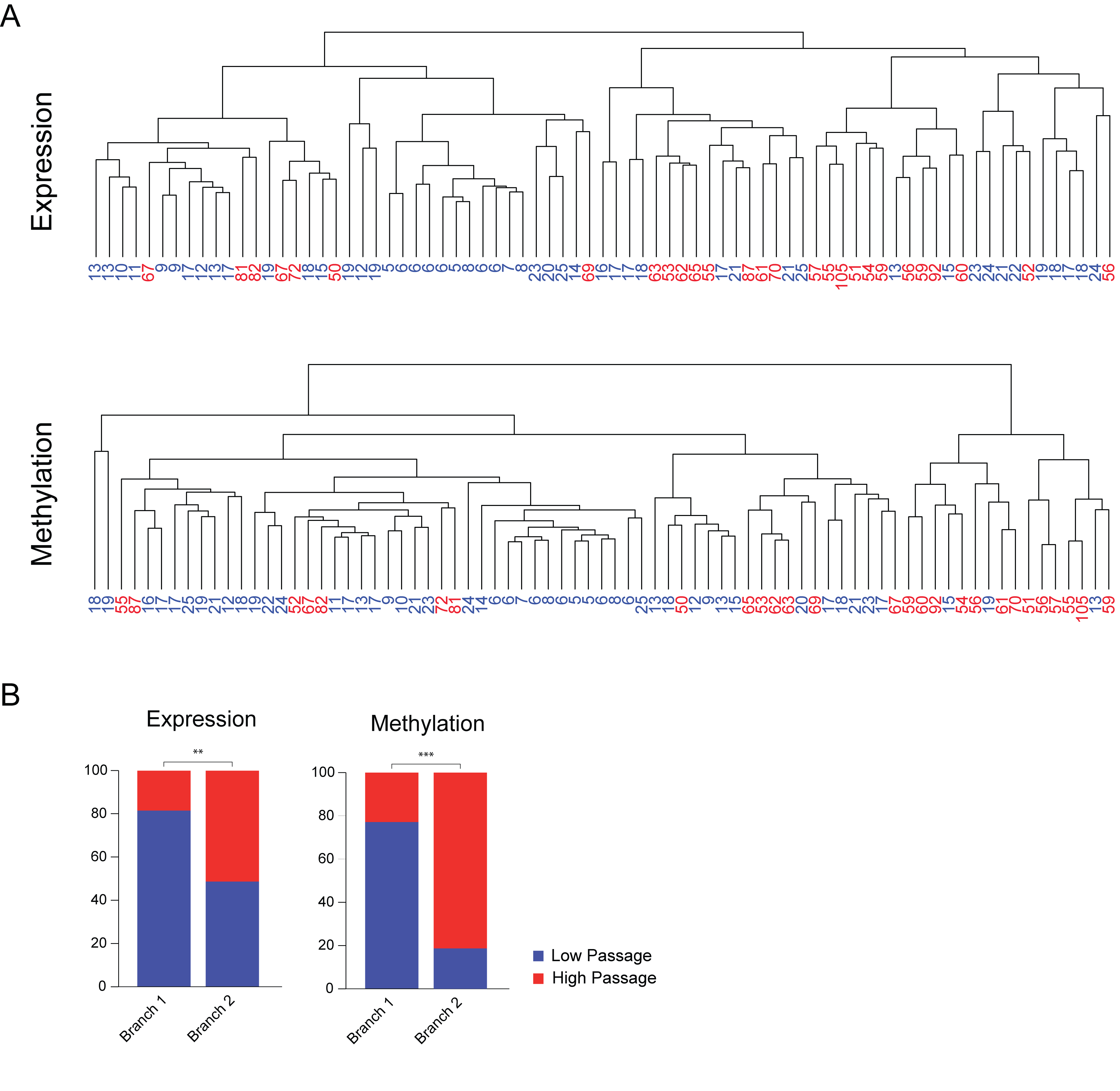

Supplement: S4 Fig — (A) Hierarchical clustering of expression and methylation patterns of data from Nazor et al. Number shown are the passage number of each sample. Clustering was performed using Euclidean distances and complete linkage. (B) Analysis of the hierarchical clustering presented in A. The proportion of low- and high passage samples in the two main branches of the trees are presented. P-values were calculated with Fisher’s exact test; *p<0.05, **p<0.01, ***p<0.001. (TIF) [file pgen.1006979.s004.tif]

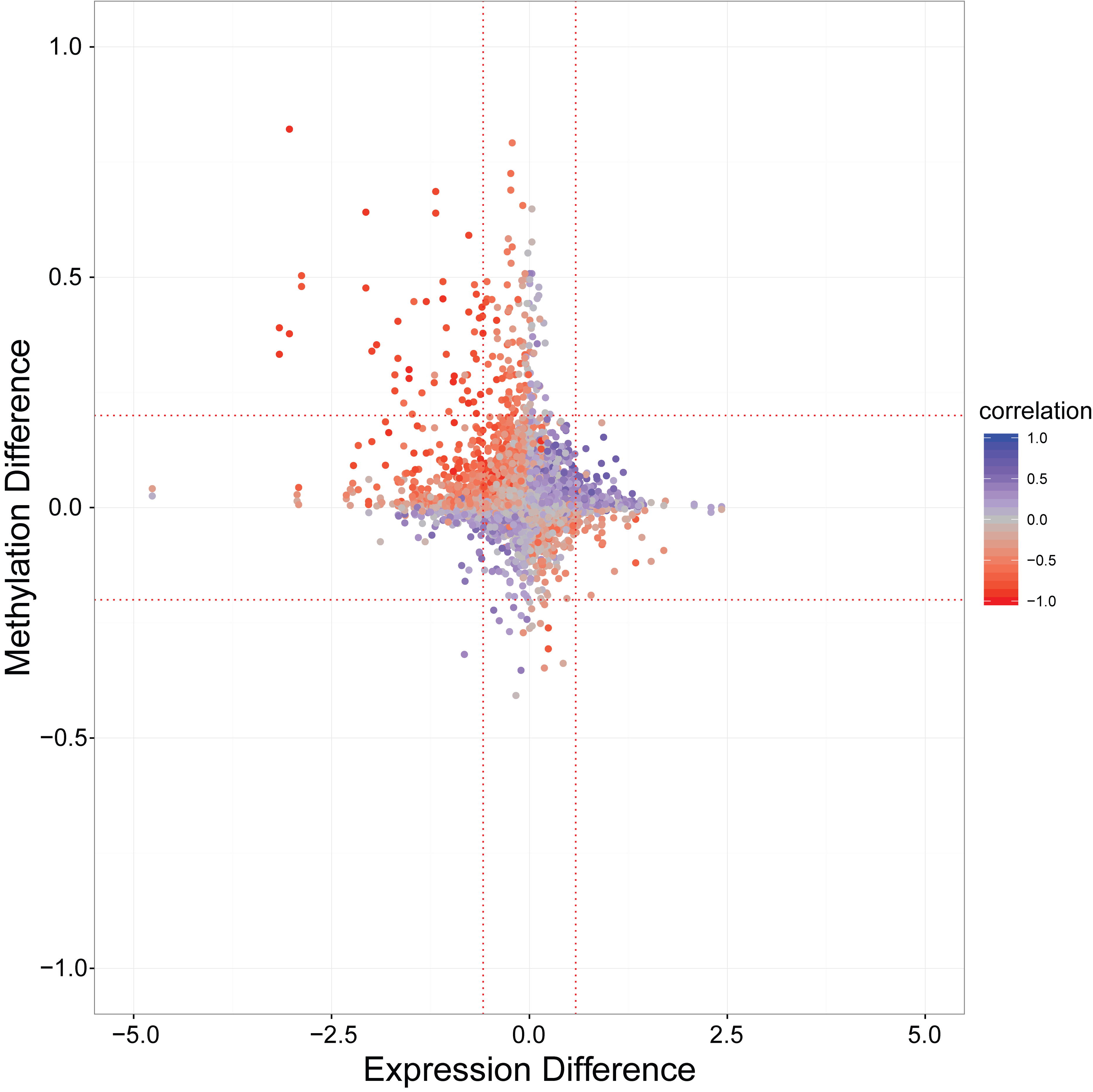

Supplement: S5 Fig — Scatter plots of expression and methylation differences between high passage and low passage samples of data from Nazor et al[15]. Here, only hESCs were included in the analysis. Values represent the change between the averages of the high passage group and the low passage group. Colors indicate the correlation between the expression and methylation. Vertical red lines represent expression change of 1.5 fold. Horizontal red lines indicated methylation change of 0.2. (TIF) [file pgen.1006979.s005.tif]

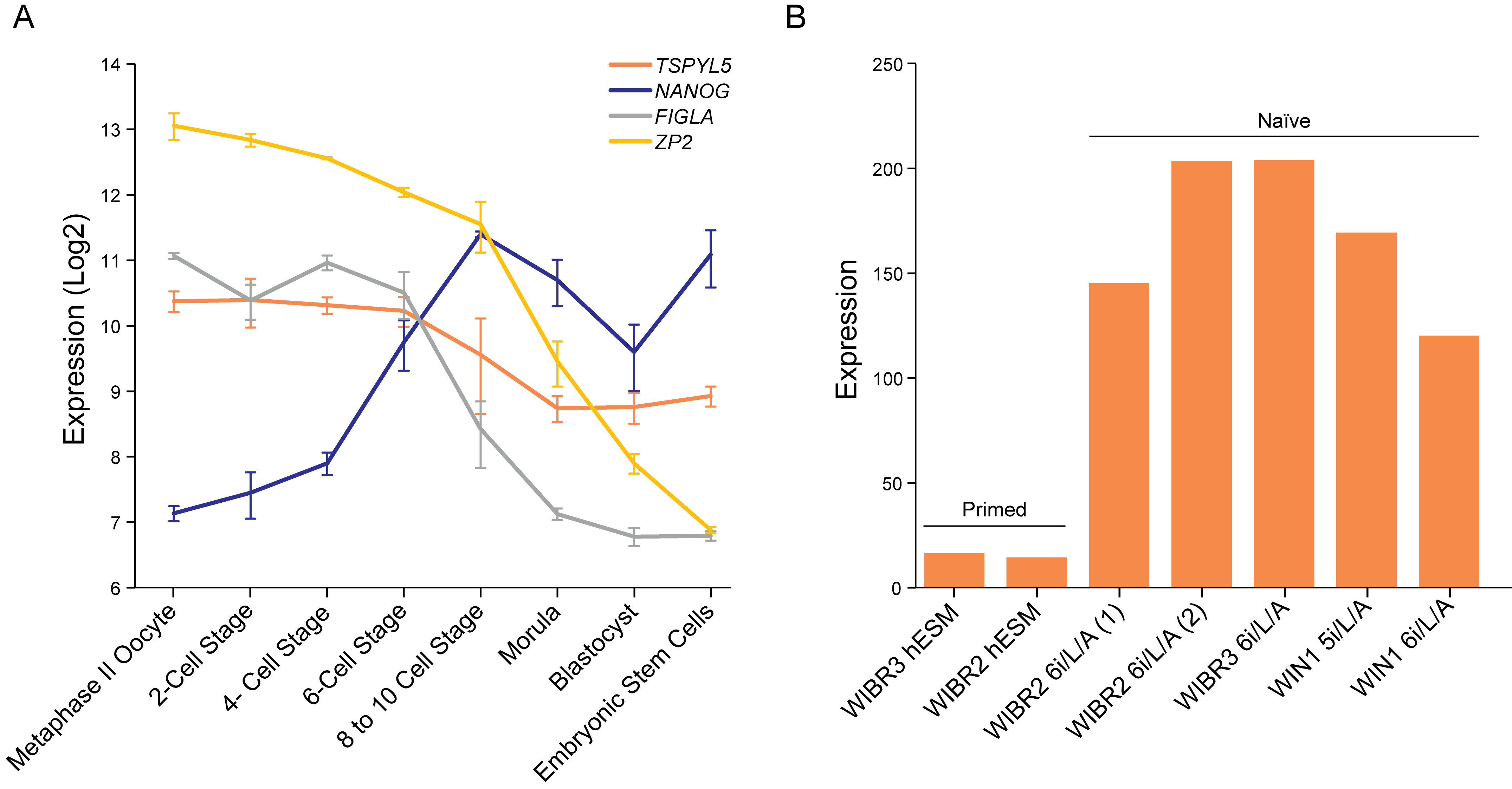

Supplement: S6 Fig — (A) Expression of two maternally deposit genes (FIGLA and ZP2), NANOG and TSPYL5 in different stages during early human development (B) TSPYL5 expression levels in hPSCs grown either with primed medium or with naïve medium[22]. (TIF) [file pgen.1006979.s006.tif]

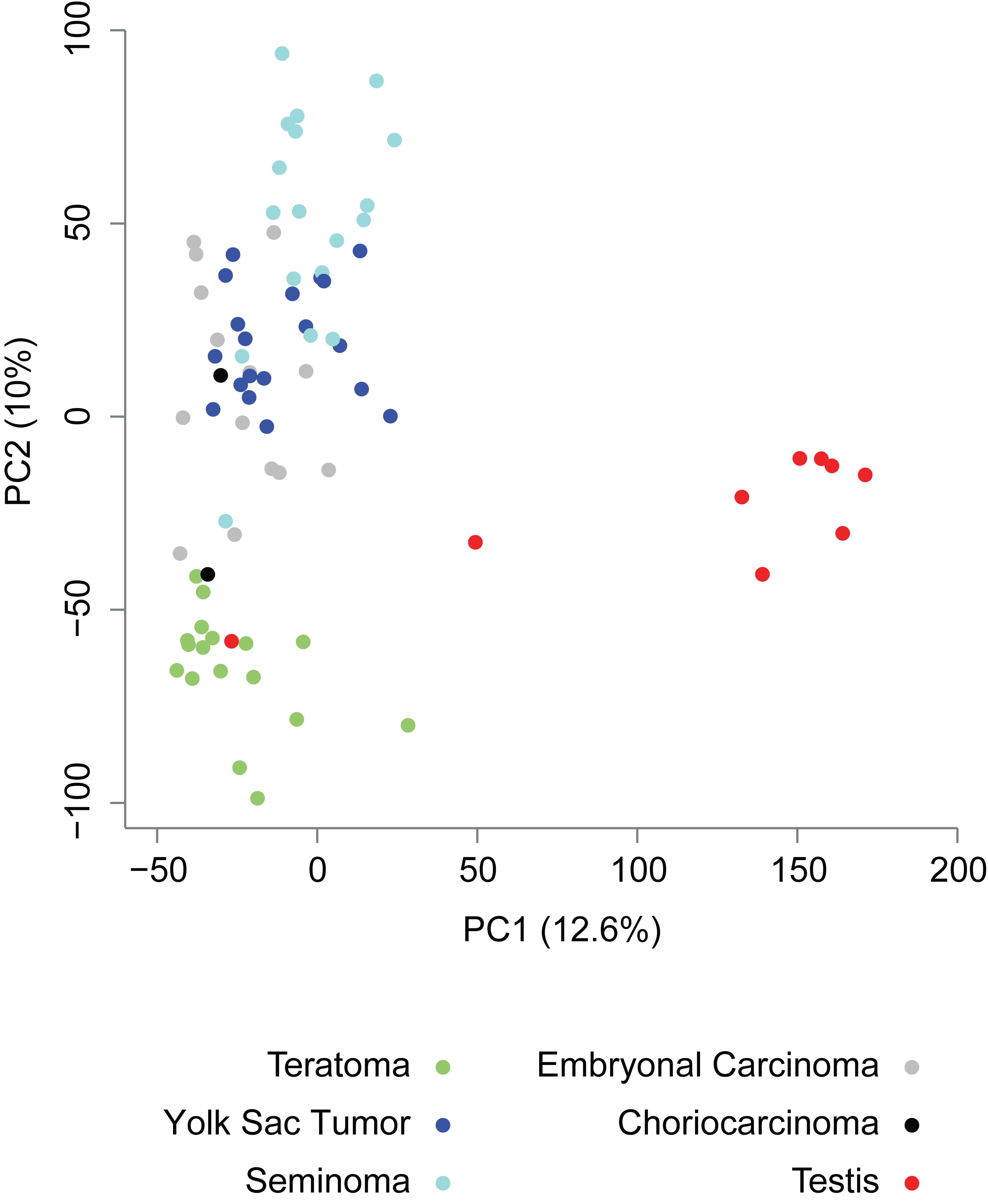

Supplement: S7 Fig — (TIF) [file pgen.1006979.s007.tif]
