# Supplementary material for: Culture-induced recurrent epigenetic aberrations in human pluripotent stem cells
Source: PLoS Genet. 2017 Aug 24;13(8):e1006979. doi: 10.1371/journal.pgen.1006979 (PMC5587343; doi:10.1371/journal.pgen.1006979)
Supplement: S1 Table — The methylation slope ranking of the 10 candidate genes, out of the total 365,146 probes, according to their position along the gene. High-ranking means high positive methylation slope, low ranking means negative methylation slope. (DOCX) [file pgen.1006979.s008.docx]

|  | **Island** | | **N_Shelf** | **N_Shore** | | **S_Shelf** | **S_Shore** | | **Undefined** | |
| --- | --- | --- | --- | --- | --- | --- | --- | --- | --- | --- |
| **TSPYL5** | 106  134  138  144 | 528  856  1695  13837 | 191001 | 189349  249633  333279 | |  | 6152  10984  38843 | |  | |
| **ZNF667** | 127  353  357  400  534  638 | 815  817  1352  3678  3879  363555 |  | 24363 | |  | 615 | | 344111  345200 | |
| **CAT** | 60  82  97  100  182  249 | 347  397  427  474  1202 |  | 2962 | | 243899 | 3862 | | 45316 | |
| **CTSF** | 123  464  1875 | 3213  4177 | 93686 | 148323 | |  | 713  11756  15669 | 209212  316335 | 159712 | |
| **MNS1** | 581  586  629 | 803  2304  2865 |  | 1415 | |  | 2857  2896  3028 | 4360  5593  159220 | 336772 | |
| **PON3** | 210  229  230  233  241  254  277  334 | 662  799  874  928  1037  1269  1509  2195 | 341109 | 13479  77776 | |  | 1333  3156  4560  5095  33651 | 311024  43690  74098  163377 |  | |
| **COX7A1** | 235  305  1392 |  |  |  | |  | 710  79859  106392 | |  | |
| **ECHDC3** | 424  619  4005 | |  | 1061  1153  1312  1798 | 2518  25706  26679  30702 | 169589 | 51174 | | 127758  129415  151485  171816 | 215733  313003  315305  328263 |
| **CTHRC1** | 481  486  521  527  669 | 2777  2919  3765  6638 |  | 39539  141523 | | 19694 | 24959  25387  333117 | |  | |
| **ACOT4** | 649  819  1310 | 5796  5995  3751 |  | 109686  170611  187019  318088 | |  | 10727  16043 | |  | |
