# Supplementary material for: Culture-induced recurrent epigenetic aberrations in human pluripotent stem cells
Source: PLoS Genet. 2017 Aug 24;13(8):e1006979. doi: 10.1371/journal.pgen.1006979 (PMC5587343; doi:10.1371/journal.pgen.1006979)
Supplement: S2 Table — (DOCX) [file pgen.1006979.s009.docx]

| **Up Regulated Genes** | | |
| --- | --- | --- |
| Gene Set Name | p-value | FDR q-value |
| GO_PROTEIN_COMPLEX_SUBUNIT_ORGANIZATION | 3.05E-07 | 0.0017 |
| GO_CHROMATIN_ASSEMBLY_OR_DISASSEMBLY | 1.08E-06 | 0.00212 |
| GO_DNA_PACKAGING | 1.69E-06 | 0.00212 |
| GO_PROTEIN_COMPLEX_ASSEMBLY | 1.9E-06 | 0.00212 |
| GO_PROTEIN_COMPLEX_BIOGENESIS | 1.9E-06 | 0.00212 |
| GO_PROTEIN_DNA_COMPLEX_SUBUNIT_ORGANIZATION | 3.8E-06 | 0.0034 |
| GO_DNA_REPLICATION_DEPENDENT_NUCLEOSOME_ASSEMBLY | 4.87E-06 | 0.0034 |
| GO_DNA_REPLICATION_DEPENDENT_NUCLEOSOME_ORGANIZATION | 4.87E-06 | 0.0034 |
| GO_PROTEIN_HETEROOLIGOMERIZATION | 5.7E-06 | 0.00353 |
| GO_CHROMATIN_SILENCING_AT_RDNA | 7.6E-06 | 0.00383 |
| GO_PROTEIN_HETEROTETRAMERIZATION | 8.25E-06 | 0.00383 |
| GO_CELLULAR_MACROMOLECULAR_COMPLEX_ASSEMBLY | 8.74E-06 | 0.00383 |
| GO_DNA_CONFORMATION_CHANGE | 8.92E-06 | 0.00383 |
| GO_MACROMOLECULAR_COMPLEX_ASSEMBLY | 1.05E-05 | 0.00417 |
| GO_POSITIVE_REGULATION_OF_GENE_EXPRESSION_EPIGENETIC | 7.23E-05 | 0.0269 |
| GO_PROTEIN_OLIGOMERIZATION | 8.12E-05 | 0.0283 |
| GO_PROTEIN_HETERODIMERIZATION_ACTIVITY | 0.000116 | 0.038 |
| GO_REGULATION_OF_CELL_DIFFERENTIATION | 0.000123 | 0.0382 |
| GO_CHROMATIN_SILENCING | 0.00013 | 0.0382 |
| GO_TELOMERE_ORGANIZATION | 0.00017 | 0.0474 |
|  |  |  |
|  |  |  |
| **Down Regulated Genes** | | |
| Gene Set Name | p-value | FDR q-value |
| GO_NEURON_PROJECTION_DEVELOPMENT | 7.38E-06 | 0.0216 |
| GO_NEURON_DIFFERENTIATION | 9.48E-06 | 0.0216 |
| GO_CELL_MORPHOGENESIS_INVOLVED_IN_NEURON_DIFFERENTIATION | 1.67E-05 | 0.0216 |
| GO_RESPONSE_TO_EXTERNAL_STIMULUS | 2.12E-05 | 0.0216 |
| GO_NEURON_PROJECTION_MORPHOGENESIS | 2.56E-05 | 0.0216 |
| GO_CELL_DEVELOPMENT | 2.68E-05 | 0.0216 |
| GO_NEURON_DEVELOPMENT | 2.72E-05 | 0.0216 |
| GO_NEURON_PROJECTION_GUIDANCE | 0.000031 | 0.0216 |
| GO_REGULATION_OF_ODONTOGENESIS_OF_DENTIN_CONTAINING_TOOTH | 4.04E-05 | 0.0251 |
| GO_CELL_MORPHOGENESIS_INVOLVED_IN_DIFFERENTIATION | 0.000081 | 0.0452 |
| GO_STRUCTURAL_CONSTITUENT_OF_CYTOSKELETON | 9.32E-05 | 0.0473 |
| GO_MOVEMENT_OF_CELL_OR_SUBCELLULAR_COMPONENT | 0.000104 | 0.0484 |
